# Human Papillomavirus Infection and Transmission Among Couples Through Heterosexual Activity (HITCH) Cohort Study: Protocol Describing Design, Methods, and Research Goals

**DOI:** 10.2196/11284

**Published:** 2019-01-16

**Authors:** Mariam El-Zein, François Coutlée, Pierre-Paul Tellier, Michel Roger, Eduardo L Franco, Ann N Burchell, HITCH Study Group

**Affiliations:** 1 Division of Cancer Epidemiology Gerald Bronfman Department of Oncology McGill University Montreal, QC Canada; 2 Centre Hospitalier de l’Université de Montréal Département de Microbiologie Médicale et Infectiologie Université de Montréal Montreal, QC Canada; 3 Department of Family Medicine McGill University Montreal, QC Canada; 4 Li Ka Shing Knowledge Intitute Department of Family and Community Medicine and Centre for Urban Health Solutions St. Michael’s Hospital Toronto, ON Canada; 5 Department of Family and Community Medicine and Dalla Lana School of Public Health University of Toronto Toronto, ON Canada

**Keywords:** human papillomavirus, HPV transmission, longitudinal study, sex partners, young adults

## Abstract

**Background:**

Human papillomavirus (HPV) epidemiological research has generally been individual based, typically focusing on women, with couple-based research mostly consisting of cross-sectional assessment of prevalent HPV infection in both partners.

**Objective:**

The HPV Infection and Transmission among Couples through Heterosexual activity (HITCH) study was set up to investigate the transmissibility of HPV among young, recently formed couples in Montreal, Canada. This paper provides an overview of the HITCH cohort study design and procedures as well as a narrative summary of the most important findings.

**Methods:**

HITCH is a longitudinal investigation of HPV transmission in recently formed heterosexual partnerships initiated within 6-month pre-enrollment, a time at which considerable transmission is believed to occur. A total of 549 newly formed dyads were recruited (2005-2011) from postsecondary institutions, including 502 young women and their male partners. An additional 46 males were enrolled at follow-up, as some women enrolled a subsequent partner at follow-up. Women aged 18-24 years were followed for 24 months for acquisition of HPV types not present at enrollment, whereas men returned for a single follow-up visit at month 4, for a sum total of 3361 clinic visits. The last follow-up visit occurred in January 2014. Extensive sociodemographic, sexual behavioral, and medical history data were collected every 2-4 months using computer-assisted, self-administered questionnaires. Furthermore, participants provided genital, blood, oral, and hand specimens for HPV assessment.

**Results:**

Although in its early analysis stage, HITCH has produced important publications. Findings from HITCH have increased the available knowledge about the natural history of HPV transmission and its determinants, provided further evidence regarding oral-oral and oral-genital routes of HPV transmission, and supplied empirically valid epidemiological parameters of HPV transmission to assist mathematical modelers in health economic assessments. In addition, HITCH data were made available to several multistudy collaborations evaluating new HPV detection assays and evidence for-or-against HPV type replacement following the introduction of HPV vaccination.

**Conclusions:**

HITCH will continue to offer a unique resource for research on HPV transmission.

**International Registered Report Identifier (IRRID):**

RR1-10.2196/11284

## Introduction

Human papillomavirus (HPV) is the most common sexually transmitted infection and a necessary cause of all cervical cancer cases in the world [1,2]. Most sexually active persons acquire it over their lifetime [3]. Although HPV is transmitted between sexual partners, the vast majority of HPV epidemiological research has been individual based and typically focused on women. Far less research has been conducted among men or couples. Couple-based HPV research has mostly consisted of cross-sectional assessment of prevalent HPV infection in both partners [4,5]. Study populations included attendees at sexually transmitted infections clinics [6], couples being evaluated for infertility [7], women referred for colposcopy and their partners [8,9], and—within the context of retrospective case-control studies—women with cervical intraepithelial neoplasia or cervical cancer [10-12]. Because most HPV infections are transient [13], infections may have cleared in one or both partners by the time couples have been together for years. To study HPV transmission, one would ideally recruit relatively young couples that are newly forming.

Initiated in 2005, the overarching aim of HPV Infection and Transmission among Couples through Heterosexual activity (HITCH) cohort study was to further our understanding of the transmissibility of HPV among recently formed couples to better inform prevention strategies. When it was developed, no other study had specifically targeted newly forming couples, and some had excluded couples of <6-month duration [10,11]. Research aims were (1) to describe the prevalence and type-specific concordance of HPV infection between young women and men in heterosexual couples; (2) to characterize and quantify risk factors for incident HPV infection among young women and men; (3) to estimate rates of male-to-female and female-to-male HPV transmission; (4) to identify behavioral risk factors and biological determinants of HPV transmission upon exposure; and (5) to characterize HPV infection in the oral tract and fingers, and their agreement with genital infection and partner’s infection status.

The HITCH cohort study (referred to hereafter as HITCH) was conducted by a team of researchers at McGill University, led by two of the authors (ELF and ANB), in collaboration with the McGill Student Health Services Clinic, Concordia Health Services Clinic, and Centre Hospitalier de l’Université de Montréal, all located in the province of Québec, Canada. The study was conducted in accordance with the principles and articles stipulated by the Tri-Council Policy Statement Ethical Conduct For Research Involving Humans [14]. It received ethical approval from institutional review boards at McGill University, Concordia University, and Université de Montréal. All participating couples provided written informed consent.

## Methods

### Study Population and Recruitment

The inclusion criteria for women were as follows: those aged 18-24 years, enrolled at a university or junior college in Montreal, intending to remain in Montreal for the next 2 years, currently heterosexually active with a male partner with whom sexual activity was initiated within the previous 6 months, willing to comply with follow-up, having an intact uterus and no history of cervical lesions or cancer, and neither currently pregnant nor planning to become pregnant in the next 24 months. Men were eligible to participate if they were aged ≥18 years and willing to comply with follow-up.

Recruitment was achieved through printed promotional materials and electronic advertisements on campuses and venues frequented by students. Interested couples were invited to visit the study website and contact study nurses.

### Study Design and Procedures

**Figure 1:**
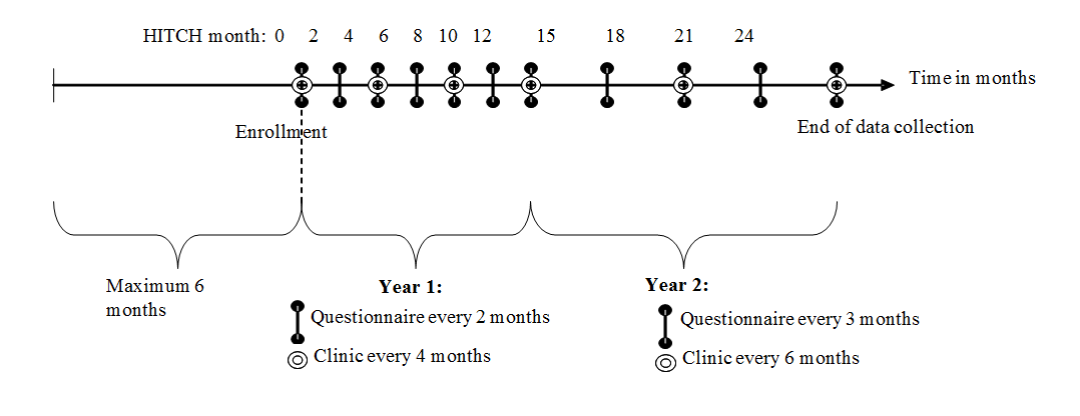
Time points and timeline for follow-up of female human papillomavirus infection and transmission among couples through heterosexual activity (HITCH) study participants.

Couples were recruited for assessment of sexual histories and HPV testing (Figure 1). Women were followed for 24 months for acquisition of HPV types not present at enrollment. Added in October 2006, men returned for a single follow-up visit at month 4. The last follow-up visit occurred in January 2014.

Men and women self-completed separate Web-based questionnaires. Women attended a clinic visit at enrollment and 5 other follow-up visits, whereas men only attended a clinic visit at enrollment and at 4 months.

Extensive efforts were made to prevent attrition, including prescheduled appointments, timely electronic reminders between administered questionnaires, in-person motivation by the research nurse at each visit, and monetary compensation (Can $50 per visit and Can $10 per questionnaire completed by a woman between visits). Reflecting in part the mobile nature of student populations, female attrition rates were 43.0% (216/502) by month 24; 48 women attended 1 visit (baseline) only, 38 attended 2, 38 attended three, 46 attended 4 and 44 women attended 5 visits. Among men who consented to return for a follow-up visit, 22.6% (124/548) were lost to follow-up (113/502 for the first male sexual partner and 11/42 for the second male sexual partner). No statistically significant differences were noted between participants who dropped out and those who remained in terms of age, smoking status, lifetime number of sex partners, age at sexual debut, or HPV status.

### Data Collection and Available Data

Information was collected using Web-based self-completed questionnaires at each visit, and is described herein in accordance with the Checklist for Reporting Results of Internet E-Surveys guidelines [15]. A secure, confidential, study- designated internet site provided participants with protected access to computerized questionnaires by assigned log-in names and passwords. In addition, 4 questionnaire versions were used: a female induction (baseline), a male induction, a female follow-up, and a male follow-up (see Multimedia Appendices 1-4). Women also completed questionnaires between visits from a computer of their choice, such that each completed 11 questionnaires in total. These questionnaires used customized text to refer to specific dates or partners to personalize questions and improve recall. Skip patterns were programmed so that respondents need only answer applicable questions. Respondents had the option of leaving responses blank if they preferred not to answer, but a warning screen appeared to ensure that no question was left blank accidentally. Table 1 shows the main topics addressed in the questionnaires and corresponding variables.

We collected biological specimens at each visit (Multimedia Appendix 5). Participants were asked to abstain from oral, vaginal, or anal sex for 24 hours before the clinic visit. At each visit, men and women provided a blood sample for HPV antibody testing. Women provided a self-collected vaginal sample [16]. For men, the examining nurse collected a specimen of epithelial cells from the penis and scrotum [17]. Beginning in 2008, we assessed oral and hand HPV infection at visits for which both partners attended (enrollment visit, 4-month follow-up visit, and subsequent follow-up visits when women enrolled a new male partner and the accompanying follow-up visit for that new male partner). Participants provided oral rinses (using a soft toothbrush and mouthwash) and a specimen of epithelial cells from the dominant hand (swabs of the index and middle fingers, and nails).

Genital, oral, and hand specimens were tested using a polymerase chain reaction (PCR) protocol based on the amplification of a 450-bp segment in the L1 HPV gene using the Linear Array HPV genotyping assay (Roche Molecular Systems) [18]. Using this technique, 36 mucosal HPV genotypes can be detected: HPVs 6, 11, 16, 18, 26, 31, 33, 34, 35, 39, 40, 42, 44, 45, 51, 52, 53, 54, 56, 58, 59, 61, 62, 66, 67, 68, 69, 70, 71, 72, 73, 81, 82, 83, 84, and 89. In addition, a molecular variant analysis of HPV16 isolates was done to obtain a more refined taxonomic level of detail. The PCR sequencing method used primers flanking a segment of the long control region of HPV [19-22]. All genital specimens found to be HPV DNA-positive for types 16, 18, 31, 33, 45, and 52 were retested using a quantitative, real-time PCR to measure type-specific viral load using specific primers [23-25].

High-resolution PCR typing of the DQB1 and DRB1 class II loci of the human leukocyte antigen (HLA) system was performed to identify specific alleles that may help explain variations in the HPV susceptibility. HLA testing was done in DNA samples extracted from oral specimens (both male and female) of the enrollment visit or, in case these were unavailable or insufficient for analysis, using specimens from subsequent visits or genital samples. In addition, sequence-based typing with the AlleleSEQR DRB1 and AlleleSEQR DQB1 assays (Abbott Molecular Diagnostics, Inc) was used to identify all possible alleles. Locus-specific exon 2 and 3 amplicons were produced by PCR amplification and then sequenced to determine allele-specific nucleotide sequence variants. To save costs, we tested only specimens from couples with an infection transmission opportunity (ie, at least one partner positive for at least one HPV type during the course of the study).

**Table 1:** Data collected using Web-based self-completed Human papillomavirus Infection and Transmission among Couples through Heterosexual activity (HITCH) questionnaires, 2005-2011.

Domain	Baseline data	Follow-up data (updates since last interview)
Sociodemographic	Sex, date of birth, place of birth (country, Canadian province), marital status, ethnicity, education status, educational institution, highest education level, employment status, parental highest level of education, financial situation while growing up	Marital status, education status, educational institution, highest education level, employment status
Smoking	Personal history of cigarette smoking (duration, quantity, and frequency)	Initiation and continuation and cessation (quantity)
Reproductive history^a^	Age at menarche, current and previous pregnancies	Current pregnancy, pregnancy since the last interview
Lifetime sexual history	Number of sexual partners (males and females, all routes), number of sexual partners (vaginal), age at first vaginal sexual intercourse, sexual orientation	N/A^b^
Sexual activity with enrolled HITCH partner	Partner date of birth, relationship status with partner, date of first engagement in sexual activity, topics discussed together (pregnancy prevention, sexually transmitted disease prevention, sexual history, ever had a sexually transmitted disease, ever tested for sexually transmitted disease including HIV/AIDS), number of previous partners involving vaginal intercourse, presence of a sexually transmitted infection, partner circumcised, frequency of engagement in sexual activities (per week, per month), frequency of masturbation (for each), frequency of oral sex (for each), vaginal intercourse (date of first and last occurrence, frequency, ie, per week, per month), condom use (frequency, breakage or slip off, put before starting vaginal intercourse, taken off during vaginal intercourse), anal intercourse^c^	Sexual activity with HITCH partner (date of enrollment of HITCH partner^c^; date of birth of HITCH partner^c^; relationship status; date of first engagement in sexual activity; topics discussed together, similar list^c^; lifetime number of partners involving vaginal intercourse^c^; ever had a sexually transmitted infection^c^; partner circumcised^c^; number of times engaged in sexual activities since the last interview, per week, per month; frequency of masturbation, for each; oral sex, for each; vaginal intercourse, date of first and last occurrence; frequency, per week, per month; condom use regarding frequency, breakage or slip off, put before starting vaginal intercourse, taken off during vaginal intercourse; anal intercourse^d^), ongoing sexual relationship, date of end of sexual relationship with HITCH partner, engagement in sexual activity with someone other than HITCH partner
Sexual activity with other partners	Concurrent sexual activity with someone other than current partner (number of sexual partners, number of ongoing sexual partners), lifetime engagement in sexual activity only with HITCH partner	Concurrent sexual activity with someone other than HITCH partner (number of sexual partners, number of ongoing sexual partners)
Contraceptive history^a^	Lifetime use of birth control methods (intrauterine device, hormonal contraceptive, condom, spermicides, diaphragm, cervical cap, sponge, vaginal douche, natural method, withdrawing or pulling out, emergency contraception such as morning-after-pill), age at first use of hormonal contraceptive, duration of use of hormonal contraceptives (months, years), birth control methods used with HITCH partner (similar list)	Use of birth control methods (similar list to baseline data)
Medical history	Number of Pap tests done,^a^ date of last Pap test (month and year),^a^ ever had a medical condition (trichomonas genital infection, venereal warts or condylomas or papillomavirus infection, chlamydia, genital herpes, syphilis, gonorrhea, ulcers or genital sores, HIV, hepatitis B, ureaplasma hominis, vaginal yeast infection,^a^ bacterial vaginosis^a^), medical condition since the start of sexual relationship with HITCH partner, signs and symptoms since the start of sexual relationship with HITCH partner (painful or frequent urination, itching or burning sensation when urinating, blood in urine, abnormal discharge, sores in the genital area, unusually painful or heavy period,^a^ vaginal itching or burning,^a^ lower back pain not caused by physical exertion)^a^	Pap test done, date of last Pap test (month and year), medical conditions (similar list to baseline data), signs and symptoms (similar list to baseline data)
Knowledge of human papillomavirus (HPV)	Ever heard of HPV, true or false statements (HPV can cause cervical cancer in women; men can carry HPV; genital warts cause cervical cancer in women; HPV can be cured with antibiotics; a person may be infected with HPV and not know it; HPV can cause penile cancer in men; HPV causes genital herpes; condoms protect against HPV; having multiple sex partners increases one’s risk for HPV; regular Pap test can help to prevent complications from HPV; HPV is the most common sexually transmitted infection; a person can get HPV from sharing a plate or fork or glass with someone who has HPV, unprotected sexual intercourse with someone who has HPV, oral sex with someone who has HPV, kissing—with exchange of saliva—someone who has HPV, sharing a washroom or shower with someone who has HPV), chances of becoming infected with HPV, chances of developing cervical or penile cancer	
HPV vaccine	Received the HPV vaccine (received part of participation in a clinical trial, number of injections, date of last injection), likelihood of choosing to be vaccinated if offered	Received the HPV vaccine (received part of participation in a clinical trial, number of injections, date of last injection), likelihood of choosing to be vaccinated if offered

^a^Questions were only asked in female respondents’ baseline questionnaires; for men, circumcision was evaluated by the nurse during the clinic visit.

^b^N/A: not applicable.

^c^Questions were only asked in female respondents’ follow-up.

^d^Men questionnaire also asked about the number of times of anal intercourse (per week, per month) and frequency of condom use during anal intercourse.

## Results

### Study Population Characteristics

HITCH enrolled the first couple in May 2005 (McGill University site) and March 2006 (Concordia University site). It reached its enrollment target in January 2011 with a total of 1051 participants (502 women and 549 men) recruited. Of 502 women, 42 enrolled a second male partner and 4 enrolled a third male partner. In total, there were 3361 clinic visits and 4408 and 2495 person-months of follow-up among women and men, respectively. Multimedia Appendix 6 shows the characteristics of the study population.

### Findings to Date

HITCH findings, to date, suggest that HPV is highly transmissible. Presented at local, national, and international scientific conferences and meetings, the spectrum of findings regarding HPV transmission have received broad acceptance in the scientific community and considerable press coverage in international media. Results are likely to influence prevention efforts for cervical cancer and other HPV-related diseases, including behavioral strategies to reduce risk.

### Prevalence of Human Papillomavirus Infection (Baseline Data for 263 Couples)

At enrollment, similar HPV prevalence (147/263, 55.8%) was detected among women and men, but couples were not necessarily concordant for the same HPV types [26]. In nearly two-thirds of couples, at least one partner was infected with ≥1 HPV type. Current partner’s status was the most important risk factor for prevalent infection (women: odds ratio [OR] 55.2, 95% CI 38.0-80.1; men: OR 58.7, 95% CI 39.8-86.3). There was evidence for a protective effect of condoms, but protection was incomplete and stronger among men than among women.

### Type-Specific Concordance of Human Papillomavirus Infection (Baseline Data for 263 Couples)

Analysis of patterns of type-specific concordance and discordance at baseline revealed that the extent of concordance was far greater than expected and consistent with rapid transmission of HPV between partners [27]. HPV was detected in 64.3% (169/263) of couples. In 41.4% (109/263) couples, both partners harbored the same HPV type—nearly 4 times more than expected if the HPV status of partners were uncorrelated. Among type-specific HPV infections in couples for whom at least one partner was infected, 42.0% (71/169) were infected with the same type for both partners (95% CI 36%-47%). This proportion provides an estimate of the per-partnership transmission probability; it increased from 25% (18/71) among couples engaging in vaginal sex for <2 months to 68% (48/71) among those at 5-6 months.

### Genital Human Papillomavirus Transmission (Longitudinal Data for 179 Couples)

Using data from the first follow-up visit, HPV transmission rates were estimated among participants with documented sexual exposure to an infected partner in couples that were discordant on ≥1 HPV types [28]. We observed little difference between male-to-female (3.5 per 100 person-months, 95% CI 2.7-4.5) and female-to-male transmission rates (4.0 per 100 person-months, 95% CI 3.0-5.5). The transmission was relatively homogeneous across HPV genotypes, alpha species, and oncogenic risk categories. The transmission rates at follow-up were lower than those estimated at baseline. This might be explained by lower infectiousness due to clearance in the index partner, defined as the one infected with a type or types not found in the other partner at baseline (nonindex partner), and lower susceptibility in the nonindex partner due to depletion of susceptibles (ie, that people who are highly susceptible would have already acquired the infection from their partner by the time of enrollment into the study).

### Determinants of Human Papillomavirus Transmission (Baseline Data for 482 Couples)

Correlates of genital HPV infection in partnerships were identified at baseline using the couple or “dyad” as the unit of analysis, rather than the individual [29]. To the best of our knowledge, this was the first analysis of its kind. HPV was detected in 69.1% (333/482) of partnerships, with both partners being HPV-positive in 49.0% (236/482) of dyads, and an equal number of male-positive/female-negative and male-negative/ female-positive dyads (43/482, 8.9%). Consistent with the sexual network theory, HPV was more likely to be present in partnerships with (1) a greater total number of lifetime partners, hence more links to the greater sexual network of young adults in Montreal; (2) shorter intervals of time since the most recent extra-dyadic partner (ie, short “gap” lengths or concurrent partnerships); and (3) greater age gaps between the male and female partner, although only the sum total of partners remained independently associated with couple-level HPV in multivariate analysis. A novel finding was that condom use with previous partners predicted lesser likelihood of detecting HPV in the current partnership. This protective effect of condoms remained statistically significant even after adjustment for potential risk factors.

### Human Papillomavirus Infection in the Oral Tract (Baseline Data for 222 Couples)

We estimated the prevalence of oral HPV and assessed risk factors among male partners at enrollment [30]. The prevalence of oral HPV among men was 7.2% (16/222). It was higher among men who were ever smokers (12/98, 12.2%), in nonmonogamous relationships (7/39, 17.9%), had a partner with oral (2/7, 28.6%) or genital (15/130, 11.5%) HPV infection. Prevalence increased with frequency of oral sex among men whose partner had a genital infection with the same HPV type. Our results provided further evidence that oral HPV may be transmitted through either oral-oral or oral-genital routes.

### Human Papillomavirus Type Replacement

It has been hypothesized that following a reduction in HPV vaccine-targeted genotypes, an increase in the prevalence of other types might occur due to reduced competition during natural infection. Any apparent postvaccination increase must be distinguished from diagnostic artifacts consequent to consensus PCR assays failing to detect HPV types present in low copy numbers in coinfected specimens. The assumption is that with a decline in vaccine-preventable types, there might be increased detection of previously “masked” types. Using data from 6 epidemiological studies, including HITCH, a total of 1200 anogenital specimens (blinded to HPV status) were reanalyzed to evaluate unmasking of HPV52 that might have been caused by elimination of HPV16 [31]. Results indicated that diagnostic artifacts due to masking may occur in some settings in the evaluation of HPV type replacement.

### Human Papillomavirus Acquisition and Clearance According to Infection Status With Vaccine-Targeted Types

We used data of 3200 females from 3 cohort studies, including HITCH [32]. Females infected with vaccine-targeted types were at a higher risk of acquiring additional HPV types and at equal risk of clearing existing infections. For example, females infected with HPV16 were at a higher risk of acquiring other alpha-9 HPV types (hazards ratio [HR] 1.9, 95% CI 1.2-3.0) but at a similar risk of clearing existing ones (HR 0.9, 95% CI 0.7-1.3). Our findings suggest that HPV type competition does not exist and type replacement is unlikely to occur.

### Natural Human Papillomavirus Type Competition in Unvaccinated Females

Pooling data from 5 prevaccination epidemiological studies, including HITCH, and applying a hierarchical Bayesian regression approach that uses shrinkage and adjustment for confounders (ie, age and lifetime number of sex partners) and other HPV types, we found HPV16 to be the most common type (prevalence range 1.0%-13.8%) [33]. HPV types were more likely to be detected as part of a multiple infection than as single infections. We did not find evidence for natural HPV type competition and type replacement.

### Y Chromosome Detection and Concordance of Genital Human Papillomavirus Infection (Baseline Data for 494 Couples)

HPV DNA detection may not always represent true infections but maybe depositions from infected sexual partners. We examined whether sexual risk factors and a biomarker, Y chromosome DNA, were associated with genital HPV partner concordance using baseline genital specimens [34]. HPV DNA Y chromosome DNA predicted type-specific HPV concordance in univariate analyses; however, in multivariable models, the independent predictors of concordance were days since the last vaginal sex and condom use. We estimated that 14% of HPV DNA detections in genital samples could be attributable to vaginal sex in the past week, which has implications not only for couple-based studies but also for individual-based HPV studies.

### Y Chromosome Detection as a Biomarker for Recent Sexual Activity (Baseline Data for 494 Couples)

We observed that this biomarker may serve as a measure of recent condomless vaginal sex in HPV studies lacking data on sexual behavior, such as surveillance studies of HPV prevalence [35]. Among female participants, detection of Y chromosome DNA in the vaginal tract decreased from 76.6% (85/111) in women who reported vaginal sex 0-1 day ago to 13% (3/23) in women whose last reported encounter was ≥15 days ago. Self-reported condom use frequency was highly and negatively correlated with Y chromosome DNA detection.

### Assortativity and Mixing by Sexual Behaviors and Sociodemographic Characteristics (Baseline Data for 502 Couples)

Sexual mixing refers to whom individuals choose as sexual partners. It is described as assortative on a characteristic if individuals have the tendency to choose partners who are similar to them on that particular characteristic (eg, age), and disassortative if individuals tend to choose partners who are different from them (eg, gender). Among 502 young adults in a new heterosexual dating partnership, we found a moderate to strong assortativity on partners’ demographic characteristics, as well as on their number of sexual partners, sexual partner acquisition rates, concurrency, and gap lengths between partnerships [36]. These findings are particularly useful for developing models of sexually transmitted infections.

### Generic Human Papillomavirus Probe Assay

The utility of a generic HPV probe assay was compared with the commercial Linear Array (Roche Diagnostics, Laval, Canada) for the detection of HPV DNA in a multistudy collaboration [37]. HITCH vaginal swabs, penile and scrotal scrapings, and fingertip brushings were among the 1013 clinical specimens analyzed. The sensitivity, specificity, and negative predictive value of the assay were 99.5% (95% CI 98.4%-99.9%), 58.6% (95% CI 53.9%-63.1%), and 98.9% (95% CI 96.5%-99.8%), respectively. Thus, the generic assay conveniently identified HPV-positive specimens.

## Discussion

### Strengths and Limitations

There are two unique features of HITCH that are novel for HPV research. The cohort is the first large-scale study of HPV acquisition involving sexual partners. Previous studies had typically sample sizes of ≤100 couples and some had as few as 25 couples [6,38,39]. More importantly, it is the only study to restrict enrollment to couples in a new sexual relationship, a time at which considerable transmission is believed to occur. Other methodological strengths of HITCH are its prospective approach, interdisciplinary framework, detailed level of assessment and wealth of covariate information, rich collection of biological specimens, and analyses of clinical samples performed according to the latest recommendations and technological advancements. Moreover, the use of frequent self-completed electronic questionnaires contributed to the collection of valid and reliable data on substantive, sensitive topics especially those relating to sexual activity.

HITCH is one of the few studies that could provide empirical estimates of HPV transmission parameters to be used in models for assessing the population health impact and cost-effectiveness of vaccination strategies. There has been an increased awareness about HPV and its role in cervical cancer since the introduction of the first prophylactic HPV vaccine in 2006, 1 year after the initiation of HITCH. Gardasil (Merck, Whitehouse Station, NJ, USA) is a quadrivalent vaccine to prevent genital warts caused by genotypes HPV6 and HPV11, as well as anal, cervical, vulvar, and vaginal cancers caused by HPVs 16/18. Approved in 2009, Cervarix (GlaxoSmithKline Biologicals, Rixensart, Belgium) is a bivalent vaccine against cervical precancerous lesions and cancer caused by HPVs 16/18, which represent 70% of all cervical cancer cases. In 2014, Gardasil 9 (Merck) vaccine was introduced to help prevent against diseases associated with HPV6, 11, 16, 18, 31, 33, 45, 52, and 58 [40].

In the postvaccine era, transmission studies such as HITCH are especially important to inform all possible prevention strategies. First, there are still some cancer caused by other high-risk HPV types, and the duration of immunity is unknown. Second, the benefits of HPV vaccination in young female adolescents will take years before being realized. Third, many teenage girls and women will still be susceptible to infection because vaccination outside of the government-funded program is only available privately at a high cost. Even for girls who may now receive the vaccine free-of-charge, implementation has not occurred without debate [41-43]. If high vaccine coverage cannot be achieved, there will be much continued transmission of the vaccine-preventable HPV types within these adolescent cohorts.

A number of limitations of this study have to be underscored. These include loss to follow-up, volunteer nature of self-selected subjects limiting the generalizability, the unavailability of hand and oral specimens for all participants at all time points, and absence of anal specimens. In addition, the potential for self-report response bias needs to be acknowledged. However, the use of self-completed electronic questionnaires to collect repeated measurements of behavioral data was likely to reduce the potential for social desirability bias. If we were to re-establish HITCH, we would have opted to (1) actively follow men at all visits; (2) collect hand, oral, and anal specimens for both men and women throughout follow-up; (3) collect data on lifetime and recent number of oral sex partners; (4) not perform serology at each visit, as this might have influenced compliance rates; and (5) instruct participants to abstain from sexual activity for a minimum of 48 hours before specimen collection to prevent potential false-positive findings.

### Future Plans

Ongoing areas of active analysis and manuscript preparation, as well as planned investigations, from our extensive program of research with HITCH data include (1) influence of HLA types on HPV type-concordance and transmission; (2) hand-genital patterns of HPV type-specific auto-concordance and couple-concordance; (3) assessment of the association between circumcision and condom use and distribution, concordance, and transmission of HPV genotypes in genital specimens; (4) impact of HPV vaccination on the prevalence, concordance, and transmission of type-specific HPV infections; (5) concordance and transmission of specific variants within HPV genotypes; (6) transmissibility of HPV focusing on the viral load and type-specific concordance of HPV infection between young couples; (7) evaluation of HPV acquisition in women for whom HPV positivity was measured both before and after the first sexual activity with a new male sex partner; (8) development and validation of a rapid, easy-to-perform and inexpensive serologic noncompetitive, multiplexed Luminex- based platform to measure total immunoglobulin G antibody; and (9) association between type-specific serological antibodies and HPV prevalence, acquisition, and clearance.

### Collaboration

Information about HITCH findings and publications is available at the McGill University Division of Cancer Epidemiology website [44]. A regularly updated list of peer-reviewed scientific publications from HITCH is available [45]. Our team is open to collaboration and data sharing with other research groups. So far, we have contributed HITCH data to multistudy collaborations [31-33,37]. Researchers interested in collaboration or further details are invited to contact the principal investigator, ELF.

## Appendix

Multimedia Appendix 1 Female induction questionnaire.

Multimedia Appendix 2 Female follow-up.

Multimedia Appendix 3 Male induction questionnaire.

Multimedia Appendix 4 Male follow-up.

Multimedia Appendix 5 Biological specimens in the HITCH Cohort Study, 2005-2011.

Multimedia Appendix 6 Baseline characteristics of the HITCH Cohort Study population (N=502), Montreal, Quebec, 2005-2011.

## Abbreviations

HITCH: Human papillomavirus Infection and Transmission among Couples through Heterosexual activity
HLA: human leukocyte antigen
HPV: human papillomavirus
HR: hazards ratio
OR: odds ratio
PCR: polymerase chain reaction
