# Supplementary material for: Human Papillomavirus Infection and Transmission Among Couples Through Heterosexual Activity (HITCH) Cohort Study: Protocol Describing Design, Methods, and Research Goals
Source: JMIR Res Protoc. 2019 Jan 16;8(1):e11284. doi: 10.2196/11284 (PMC6352011; doi:10.2196/11284)
Supplement: Multimedia Appendix 2 [file resprot_v8i1e11284_app2.pdf]

HITCH

FEMALE RESPONDENT
FOLLOW-UP

Thank you very much for returning to complete your next survey for the HITCH Cohort Study. Your assistance will ensure that the study will be able to answer questions about how HPV is transmitted, how much risk there is after a sexual encounter, and what women and men can do to protect themselves.

This follow-up survey will ask questions about you, your current health, and recent sexual behaviour. It should take about 20 minutes to complete. Please use a pencil to write your answers. Most questions require that you simply circle the response that applies to you. Other questions ask for a specific answer, such as a date or another number. Depending on your answer for some questions, you may be told to skip past some questions or go to a different part of the questionnaire. Please read these skip instructions carefully. They are to save you time so that you won't have to answer questions that do not apply to you.

Many questions refer to the time since you filled out your last survey for HITCH. According to our records, your last survey was completed on _______________ **(dd/mm/yy)**. A number of questions will ask about the male partner who enrolled in HITCH with you. Please refer to him for all questions that mention your "HITCH partner". However, if you invited a new partner to enroll after you had already enrolled in the study, then please refer only to this most recent partner as your "HITCH partner".

There are no right or wrong answers to any question. Since we will be using this survey with many people with different experiences, you may find that some of the questions do not seem to apply to you. Other questions will definitely be relevant. Many questions ask you to think back over your adult years, or over the past several months, to recall specific information. Please take your time to consider each question carefully.

Remember that all your answers are completely confidential. You can leave blank any question that you do not want to answer. If you cannot possibly remember the information, you can also leave the question blank, but we encourage you to try to answer all questions. A good guess is always better than no information at all. If you would like to tell us more about any specific items, please use the available space at the end of the questionnaire.

Let's begin!

Please record your HITCH ID number, today's date, and the time you started filling out the survey here.

ID number: _______________

Today's date: _______________

Time at start of survey: _______________

General Information

The first part of the survey is to update general information about you.

1. What is your current marital status?

1	*Single/never married*
2	*Unmarried but living with a partner*
3	*Married*
4	*Divorced/separated*
5	*Widowed*

2.a)Are you presently enrolled at McGill/Concordia or at another educational institution?

0	*No*
1	*Yes (specify)*

→ ***If no, go to question 2c).***

b) How are you presently enrolled as a student?

1	*Undergraduate student*
2	*Graduate studies – Diploma, Master's, or Doctoral Program*
3	*Community college or CEGEP student*
0	*Other (e.g. Trainee, Postdoctoral studies, Sabbatical) (specify)*

c) What is the highest level of education that you have completed?

1	*No formal education*
2	*Grade 8 or less*
3	*Some high school*
4	*High school graduate*
5	*Some community college or CEGEP*
6	*Community college or CEGEP graduate*
7	*Some university*
8	*University graduate (including undergraduate, graduate and postgraduate studies)*
0	*Other (specify)*

d) What is your current employment status? Circle one only.

1	*Working full time (30 hours/week or more)*
2	*Working part time (<30 hours/week)*
3	*Not working due to full-time studies*
4	*On parental leave*
5	*Looking for work*
6	*Temporarily off sick*
7	*No longer able to work*
8	*No longer wish to work*
9	*Homemaker*
0	*Other (specify)*

Smoking Update

The following questions are about your tobacco smoking habits.

3. Since your last survey, have you smoked cigarettes?

0	*No*
1	*Yes*

→ ***If no, go to question 5.***

4. On average, how many cigarettes have you smoked a day since you your last survey? (If you smoke less than one cigarettte per day, please answer “0.5”.)

# cigarettes per day

Reproductive Update

5. To the best of your knowledge, are you currently pregnant?

0	*No*
1	*Yes*
7	*Do not know*

→ ***If yes or do not know, go to Sexual Behaviour Update on page 6.***

6. Have you been pregnant since your last survey?

0	*No*
1	*Yes*
7	*Do not know*

Sexual Behaviour Update

The next questions are about your recent sexual behaviour. Please take the time to recall this information as accurately as possible. Remember that all the information you give will be kept entirely confidential.

Throughout this survey, we will refer to various specific sexual acts. As in previous surveys, these terms are explained below so that everyone attaches the same meanings to them. Please be sure to review these definitions again. If you need any further help or explanation, please ask the Research Nurse.

partners or sexual partners:	People who have had sex together—whether once, or just a few times, or as regular partners, or as married partners
genital area:	A man's penis or a woman's vulva and vagina—that is, the sex organs
oral sex:	A man's or a woman's mouth on a partner's genital area
vaginal sex or vaginal sexual intercourse:	A man's penis in a woman's vagina. This is what most people usually think of as "having sex" or "sexual intercourse"
anal sex or anal sexual intercourse:	A man's penis in a sexual partner's anus or rectum
mutual masturbation:	Hand stimulation of a (*woman/man's*) genital area by (*his/her*) partner, NOT involving intercourse (vaginal, oral, or anal)
sexual activity:	Mutual masturbation, oral sex, vaginal sex, or anal sex
sexual intercourse:	This includes oral, vaginal, and anal sex

Sexual Activity with HITCH Partner

7. Since your last survey, did you engage in sexual activity with a partner who enrolled in HITCH? Remember that by sexual activity, we mean mutual masturbation, oral, vaginal, and/or anal sex.

0	*No*
1	*Yes*

→ ***If yes, go to question 11.***

8. Do you consider your sexual relationship with your HITCH partner to be ongoing?

0	*No*
1	*Yes*
7	*Do not know*

→ ***If yes or do not know, go to question 10.***

9. When did your sexual relationship with your HITCH partner end? (If you only know the approximate date, specify the month and year.)

dd	*mm*	*yyyy*

10. Since your last survey, did you engage in sexual activity with someone else?

0	*No*
1	*Yes*

→ ***If no, go to question 33.***

→ ***If yes, go to question 28.***

11. When did your HITCH partner enroll in the study?

1	*At the same time that I enrolled*
2	*He enrolled after I had already enrolled*

→ *If he enrolled after you had already enrolled, please specify the date he enrolled. If you only know the approximate date, specify the month and year.*

dd	*mm*	*yyyy*

12. What are your HITCH partner's initials? (If you prefer, you can use an alias or nickname for this partner. Please choose one that you will remember later.)

Initials/Alias

13. What is his date of birth?

dd	*mm*	*yyyy*

14. a) Is he your…

1	*Husband*
2	*Common-law or live-in partner (living together)*
3	*Dating partner/boyfriend*
4	*Friend*
5	*Casual acquaintance*
6	*Not sure –we just met*
0	*Other (specify)*

b) Do you consider your sexual relationship with him to be…

1	*Ongoing and steady/regular*
2	*Ongoing but sporadic/on and off*
3	*One or a few times only*
4	*Our sexual relationship was ongoing but has now ended*
0	*Other (specify)*

15. When did you first engage in sexual activity with your HITCH partner? Remember that by sexual activity, we mean mutual masturbation, oral, vaginal, and/or anal sex. (If you only know the approximate date, specify the month and year.)

dd	*mm*	*yyyy*

16. Have you and your HITCH partner ever discussed the following since the start of your sexual relationship?

	No	*Yes*	*Do not remember*
i) Pregnancy prevention	*0*	*1*	*7*
ii) Sexually transmitted disease prevention	*0*	*1*	*7*
iii) **His** sexual history	*0*	*1*	*7*
iv) **Your** sexual history	*0*	*1*	*7*
v) Whether **he** ever had a sexually transmitted disease	*0*	*1*	*7*
vi) Whether **you** ever had a sexually transmitted disease	*0*	*1*	*7*
vii) Whether **he** had ever been tested for sexually transmitted diseases (including HIV/AIDS)	*0*	*1*	*7*
viii) Whether **you** had ever been tested for sexually transmitted diseases (including HIV/AIDS)	*0*	*1*	*7*

17. To the best of your knowledge…

a) …what is the number of female partners with whom he has had vaginal intercourse in his lifetime, including you (if applicable)?

☐ *Approximate #* *OR* ☐ *Check here if do not know*

b) …has he ever had a sexually transmitted infection (e.g., chlamydia, gonorrhea, syphilis, genital herpes, pubic lice, HIV, hepatitis B)?

0	*No*
1	*Yes*
7	*Do not know*

c) …is he circumcised?

0	*No*
1	*Yes*
7	*Do not know*

The next series of questions are about sexual activities you may have engaged in with your HITCH partner since your last survey. Remember that by sexual activity, we mean mutual masturbation, oral, vaginal, and/or anal sex.

18. Since the your last survey, how many times did you engage in sexual activities with your HITCH partner?

	OR		OR	
Approximate #		*Approximate # times per week*		*Approximate # times per month*

During those sexual encounters…

19. …how often did you masturbate him?

0	*Never (0%)*
1	*Rarely (1-25%)*
2	*Some of the time (26-75%)*
3	*Most of the time (76-99%)*
4	*Always (100%)*

20. …how often did he masturbate you?

0	*Never (0%)*
1	*Rarely (1-25%)*
2	*Some of the time (26-75%)*
3	*Most of the time (76-99%)*
4	*Always (100%)*

21. …how often did you give him oral sex?

0	*Never (0%)*
1	*Rarely (1-25%)*
2	*Some of the time (26-75%)*
3	*Most of the time (76-99%)*
4	*Always (100%)*

22. …how often did he give you oral sex?

0	*Never (0%)*
1	*Rarely (1-25%)*
2	*Some of the time (26-75%)*
3	*Most of the time (76-99%)*
4	*Always (100%)*

23. a) Have you ever had vaginal intercourse with your HITCH partner?

0	*No*
1	*Yes*

If no, go to question 26.

b) When did you first have vaginal intercourse with him? (If you only know the approximate date, specify the month and year.)

dd	*mm*	*yyyy*

c) When was the last time you had vaginal intercourse with him?

dd	*mm*	*yyyy*

d)Since your last survey, how many times did you have vaginal intercourse with him?

	OR		*OR*	
Approximate #		*Approximate # times per week*		*Approximate # times per month*

24. Since your last survey, how often did you use condoms for vaginal intercourse with him? (This includes male and female condoms.)

0	*Never (0%)*
1	*Rarely (1-25%)*
2	*Some of the time (26-75%)*
3	*Most of the time (76-99%)*
4	*Always (100%)*

➔ ***If never, go to question 26.***

25. **When you used condoms** for vaginal intercourse with him since your last survey…

a) …did the condom ever break or slip off?

0	*No*
1	*Yes*
7	*Do not remember*

b) …did you always put the condom on before starting to have vaginal intercourse?

0	*No*
1	*Yes*
7	*Do not remember*

c) …did you ever take the condom off then continue to have unprotected vaginal intercourse with you?

0	*No*
1	*Yes*
7	*Do not remember*

26. Since your last survey, did you have anal intercourse with him?

0	*No*
1	*Yes*

Sexual Activity with Other Partners

The next questions are about sexual activities you may have engaged in with someone other than your HITCH partner since your last survey.

27. Since your last survey, did you engage in sexual activity with someone else? **Remember that by sexual activity, we mean mutual masturbation, oral, vaginal, and/or anal sex.**

0	*No*
1	*Yes*

→ ***If no, go to question 31.***

28. Since your last survey, how many **other** sexual partners did you have?

Approximate #

→ ***If 5 or fewer other partners, complete a yellow OP Form for each of these partners, then go to question 31 on page 15.***
<u>Do not answer questions 29-30.</u>

→ ***If more than 5 other partners, advance to question 29.***

29. Since your last survey, how many **other** sexual partners were ongoing sexual partners? That is, partners with whom you had an **ongoing sexual relationship** (e.g. dating partner, husband, common-law partner)?

Approximate #

→ ***Complete a yellow OP Form for each of these partners, then advance to question 30.***

30. Since your last survey, how many **other** sexual partners were sexual partners with whom you did **<u>not</u> have an ongoing sexual relationship**? (e.g. one-night stands or flings)?

Approximate #

→ ***Complete one orange AP Form for all of these partners combined, then advance to question 31.***

Contraceptive Update

Here we would like to know about methods of birth control or family planning that you and a partner may have used since your last survey.

31. During that time, did you use any protection to keep from getting pregnant? (For example, hormonal contraceptives, spermicides, condoms, the rhythm method.)

0	*No*
1	*Yes*

→ ***If no, go to question 33.***

32. Which of the following birth control methods have you or your partner(s) used since your last survey?

	Did not use it	*Used it but not regularly*	*Used it regularly (at least 75% of the time)*
i) Mirena, an intrauterine device (IUD) with progestin	*0*	*1*	*2*
ii) Loop, coil, or other intrauterine device (IUD) (not including Mirena)	*0*	*1*	*2*
iii) Hormonal contraceptive (e.g. birth control pill, Depo-Provera injections, vaginal ring, Norplant, the patch), not including Mirena	*0*	*1*	*2*
iv) Condom	*0*	*1*	*2*
v) Spermicide foam, jelly, cream, or suppository	*0*	*1*	*2*
vi) Diaphragm	*0*	*1*	*2*
vii) Cervical cap	*0*	*1*	*2*
viii) Sponge	*0*	*1*	*2*
ix) Vaginal douche	*0*	*1*	*2*
x) Rhythm, calendar, or natural method	*0*	*1*	*2*
xi) Withdrawal/pulling out	*0*	*1*	*2*
xii) Emergency contraception (the “morning-after pill”)	*0*	*1*	*2*

Medical Update

The next questions will update your medical history.

33. Since your last survey, have you had a Pap test?

0	*No*
1	*Yes*
7	*Do not remember*

34. When was your last Pap test?

mm	*yyyy*

☐ Check here if never had a Pap test

35. Since your last survey, did a doctor tell you that you had one of the following conditions?

	No	*Yes*	*Don't remember*
i) Trichomonas vaginal infection	*0*	*1*	*7*
ii) Venereal warts, condylomas, or papilloma virus infection	*0*	*1*	*7*
iii) Chlamydia	*0*	*1*	*7*
iv) Genital herpes	*0*	*1*	*7*
v) Syphilis	*0*	*1*	*7*
vi) Gonorrhea	*0*	*1*	*7*
vii) Ulcers or genital sores	*0*	*1*	*7*
viii) HIV	*0*	*1*	*7*
ix) Hepatitis B	*0*	*1*	*7*
x) Ureaplasma hominis	*0*	*1*	*7*
xi) Vaginal yeast infection, thrush, or candidiasis	*0*	*1*	*7*
xii) Bacterial vaginosis	*0*	*1*	*7*

36. Since your last survey, did you have any of the following signs/symptoms?

	No	Yes	Don't remember
i) Painful urination, or difficulty urinating, or frequent urination	*0*	*1*	*7*
ii) Itching or burning sensation when urinating	*0*	*1*	*7*
iii) Blood in urine	*0*	*1*	*7*
iv) Abnormal vaginal discharge (i.e. different colour, consistency, or odor)	*0*	*1*	*7*
v) Sores in the genital area	*0*	*1*	*7*
vi) Unusually painful or heavy period	*0*	*1*	*7*
vii) Vaginal itching or burning	*0*	*1*	*7*
viii) Lower back pain not caused by physical exertion	*0*	*1*	*7*

37.a) Have you received the HPV vaccine?

0	*No, never*
1	*Yes, since my last survey*
2	*Yes, but not since my last survey*
7	*Don't know*

→ ***If never or don't know, go to question 38.***

b) Did you receive the vaccine as part of participation in a clinical trial?

0	*No*
1	*Yes*
7	*Don't know*

c) How many injections of the HPV vaccine have you received, including booster shots?

#

d) When was your last injection of the HPV vaccine? (If you only know the approximate date, specify the month and year.)

dd	*mm*	*yyyy*

→ ***Go to question 39.***

38. If the HPV vaccine is offered to you in the future, how likely is it that you will choose to be vaccinated?

1	*Very likely*
2	*Somewhat likely*
3	*Neutral*
4	*Somewhat unlikely*
5	*Very unlikely*

39. Please use the space below if you have any additional information you feel would be important for us to know.

Please record the time you stopped filling out the survey here.

Time finished survey: ________________

This brings us to the end of this survey. Please take a moment to review you answers in all sections of the questionnaire. Again, try to answer all questions. A good guess will be more useful to the study than leaving the question blank.

Thank you very much for your participation!
