# Supplementary material for: Human Papillomavirus Infection and Transmission Among Couples Through Heterosexual Activity (HITCH) Cohort Study: Protocol Describing Design, Methods, and Research Goals
Source: JMIR Res Protoc. 2019 Jan 16;8(1):e11284. doi: 10.2196/11284 (PMC6352011; doi:10.2196/11284)
Supplement: Multimedia Appendix 3 [file resprot_v8i1e11284_app3.pdf]

HITCH

MALE RESPONDENT

Thank you very much for agreeing to complete this survey for the HITCH Cohort Study. Your help will ensure that the study will be able to answer questions about how HPV is transmitted, how much risk there is after a sexual encounter, and what men and women can do to protect themselves.

The survey will ask questions about you, your health and sexual history, recent sexual behaviour, and your knowledge of and attitudes toward human papillomavirus (HPV). It should take about 30 minutes to complete. Please use a pencil to write your answers. Most questions require that you simply circle the response that applies to you. Other questions ask for a specific answer, such as your age, a date, or another number. Depending on your answer for some questions, you may be told to skip past some questions or go to a different part of the questionnaire. Please read these skip instructions carefully. They are to save you time so that you won't have to answer questions that do not apply to you.

The HITCH Cohort Study enrolls couples who recently initiated a sexual relationship. A number of questions will ask about the partner who enrolled with you. Please refer to her for all questions that mention your "HITCH partner".

There are no right or wrong answers to any question. Since we will be using this survey with many people with different experiences, you may find that some of the questions do not seem to apply to you. Other questions will definitely be relevant. Many questions ask you to think back over your adult years, or over the past several months, to recall specific information. Please take your time to consider each question carefully.

Remember that all your answers are completely confidential. You can leave blank any question that you do not want to answer. If you cannot possibly remember the information, you can also leave the question blank, but we encourage you to try to answer all questions. A good guess is always better than no information at all. If you would like to tell us more about any specific items, please use the available space at the end of the questionnaire.

Let's begin!

Please record your HITCH ID number, today's date, and the time you started filling out the survey here.

ID number: ______________

Today's date: ______________

Time at start of survey: ______________

General Information

The first part of the questionnaire concerns general information about you and where you live.

1. What is your date of birth?

dd	*mm*	*yyyy*

2.a) In what country where you born?

country

b) If born in Canada, indicate province.

province

3. What is your current marital status? Please circle your answer.

1	*Single/never married*
2	*Unmarried but living with a partner*
3	*Married*
4	*Divorced/separated*
5	*Widowed*

4. The Montreal area is made up of many ethnic groups. We would like to know in which group you would place yourself. Circle the most appropriate category.

1	*French Canadian*
2	*English Canadian*
3	*Black Canadian*
4	*Aboriginal*
5	*Latin American*
6	*Greek*
7	*Italian*
8	*South Asian*
9	*East Asian*
0	*Other (specify)*

5. a) What is/was your father's (or primary male caregiver's) highest level of education that he completed?

1	*No formal education*
2	*Grade 8 or less*
3	*Some high school*
4	*High school graduate*
5	*Some community college or CEGEP*
6	*Community college or CEGEP graduate*
7	*Some university*
8	*University graduate (including undergraduate, graduate and postgraduate studies)*
0	*Other (specify)*

b) What is/was your mother's (or primary female caregiver's) highest level of education that she completed?

1	*No formal education*
2	*Grade 8 or less*
3	*Some high school*
4	*High school graduate*
5	*Some community college or CEGEP*
6	*Community college or CEGEP graduate*
7	*Some university*
8	*University graduate (including undergraduate, graduate and postgraduate studies)*
0	*Other (specify)*

c) On average, would you say that your family's financial situation while growing up was…

1	*Difficult*
2	*Moderate*
3	*Comfortable*
4	*Very comfortable*

d)Are you presently enrolled at McGill/Concordia or at another educational institution?

0	*No*
1	*Yes (specify)*

→ ***If no, go to question 5f.***

e) How are you presently enrolled as a student?

1	*Undergraduate student*
2	*Graduate studies – Diploma, Master's, or Doctoral Program*
3	*Community college or CEGEP student*
0	*Other (e.g. Trainee, Postdoctoral studies, Sabbatical) (specify)*

f) What is the highest level of education that you have completed?

1	*No formal education*
2	*Grade 8 or less*
3	*Some high school*
4	*High school graduate*
5	*Some community college or CEGEP*
6	*Community college or CEGEP graduate*
7	*Some university*
8	*University graduate (including undergraduate, graduate and postgraduate studies)*
0	*Other (specify)*

g) What is your current employment status? Circle one only.

1	*Working full time (30 hours/week or more)*
2	*Working part time (<30 hours/week)*
3	*Not working due to full-time studies*
4	*On parental leave*
5	*Looking for work*
6	*Temporarily off sick*
7	*No longer able to work*
8	*No longer wish to work*
9	*Homemaker*
0	*Other (specify)*

6. How long have you lived in Montreal?

☐ *OR* ☐ *OR* ☐

# months *#years* *Check here if do not live in Montreal*

Smoking History

The following questions are about your tobacco smoking habits.

7. Have you smoked a total of at least 100 cigarettes (4 or more packs) in your lifetime?

0	*No*
1	*Yes*

→ ***If no, go to Lifetime Sexual History on page 6.***

8. Have you ever smoked cigarettes regularly, that is, one cigarette or more each day for a year or more?

0	*No*
1	*Yes*

→ ***If no, go to Lifetime Sexual History on page 6.***

9. At what age did you start to smoke regularly?

[ ]
Age in years

10.a) Do you still smoke regularly?

0	*No*
1	*Yes*

If no → b) At what age did you stop smoking regularly?

[ ]
Age in years

11. On average, how many cigarettes have you smoked a day since you began smoking regularly? (If you have stopped smoking regularly, please consider only those periods during which you were smoking regularly).

[ ]
# cigarettes per day

Lifetime Sexual History

The next questions are about your sexual history. We realize this is a personal subject, but it is very important to the study of HPV. Please take the time to recall this information as accurately as possible. Remember that all the information you give will be kept entirely confidential.

Throughout this survey, we will refer to various specific sexual acts. These terms are explained below so that everyone attaches the same meanings to them. Please be sure to read these definitions. If you need any further help or explanation, please ask the Research Nurse.

partners or sexual partners:	People who have had sex together—whether once, or just a few times, or as regular partners, or as married partners
genital area:	A man’s penis or a woman’s vulva and vagina—that is, the sex organs
oral sex:	A man’s or a woman’s mouth on a partner’s genital area
vaginal sex or vaginal sexual intercourse:	A man’s penis in a woman’s vagina. This is what most people usually think of as “having sex” or “sexual intercourse”
anal sex or anal sexual intercourse:	A man’s penis in a sexual partner’s anus or rectum
mutual masturbation:	Hand stimulation of a (*woman/man’s*) genital area by (*his/her*) partner, NOT involving intercourse (vaginal, oral, or anal)
sexual activity:	Mutual masturbation, oral sex, vaginal sex, or anal sex
sexual intercourse:	This includes oral, vaginal, and anal sex

12.a) Please think about all the people with whom you have engaged in sexual intercourse (oral, vaginal or anal). In total, with how many people—female or male— have you engaged in sexual intercourse in your lifetime?

Approximate #

b) How many were female?

Approximate #

c) How many were male?

Approximate #

13. How old were you when you first had vaginal sexual intercourse?

Age in years *OR* *Check here if never had vaginal sexual intercourse*

→ ***If never, go to question 15.***

14. Throughout your life, what is the number of female partners with whom you have had vaginal sexual intercourse?

Approximate #

15. Do you consider yourself to be:

1	*Heterosexual/straight*
2	*Bisexual*
3	*Gay/homosexual*
0	*Other (specify)*

Sexual Activity with Enrolled HITCH Partner

The next questions are about the female partner who enrolled in HITCH with you. We will refer to her as your “HITCH partner”.

16. What are her initials? (If you prefer, you can use an alias or nickname for this partner. Please choose one that you will remember later.)

Initials/Alias

17. What is her date of birth?

dd	*mm*	*yyyy*

18.a) Is she your…

1	*Wife*
2	*Common-law or live-in partner (living together)*
3	*Dating partner/girlfriend*
4	*Friend*
5	*Casual acquaintance*
6	*Not sure –we just met*
0	*Other (specify)*

b) Do you consider your sexual relationship with her to be…

1	*Ongoing and steady/regular*
2	*Ongoing but sporadic/on and off*
3	*One or a few times only*
0	*Other (specify)*

19. When did you first engage in sexual activity with her? Remember that by sexual activity, we mean mutual masturbation, oral, vaginal, and/or anal sex. (If you only know the approximate date, specify the month and year.)

dd	*mm*	*yyyy*

20. Have you and your HITCH partner ever discussed the following since the start of your sexual relationship?

	No	*Yes*	*Do not remember*
i) Pregnancy prevention	*0*	*1*	*7*
ii) Sexually transmitted disease prevention	*0*	*1*	*7*
iii) **Your** sexual history	*0*	*1*	*7*
iv) **Her** sexual history	*0*	*1*	*7*
v) Whether **you** ever had a sexually transmitted disease	*0*	*1*	*7*
vi) Whether **she** ever had a sexually transmitted disease	*0*	*1*	*7*
vii) Whether **you** had ever been tested for sexually transmitted diseases (including HIV/AIDS)	*0*	*1*	*7*
viii) Whether **she** had ever been tested for sexually transmitted diseases (including HIV/AIDS)	*0*	*1*	*7*

21. To the best of your knowledge…

a) …what is the number of male partners with whom she has had vaginal intercourse in her lifetime, including you (if applicable)?

☐ *Approximate #* *OR* ☐ *Check here if do not know*

b) …has she ever had a sexually transmitted infection (e.g., chlamydia, gonorrhea, syphilis, genital herpes, pubic lice, HIV, hepatitis B)?

0	*No*
1	*Yes*
7	*Do not know*

The next series of questions are about sexual activities you may have engaged in with your HITCH partner since you first started your sexual relationship.

22. Since the start of your sexual relationship with your HITCH partner, how many times did you engage in sexual activities with her? Remember that by sexual activity, we mean mutual masturbation, oral, vaginal, and/or anal sex.

	OR		*OR*	
Approximate #		*Approximate # times per week*		*Approximate # times per month*

During those sexual encounters…

23. …how often did you masturbate her?

0	*Never (0%)*
1	*Rarely (1-25%)*
2	*Some of the time (26-75%)*
3	*Most of the time (76-99%)*
4	*Always (100%)*

24. …how often did she masturbate you?

0	*Never (0%)*
1	*Rarely (1-25%)*
2	*Some of the time (26-75%)*
3	*Most of the time (76-99%)*
4	*Always (100%)*

25. …how often did you give her oral sex?

0	*Never (0%)*
1	*Rarely (1-25%)*
2	*Some of the time (26-75%)*
3	*Most of the time (76-99%)*
4	*Always (100%)*

26. …how often did she give you oral sex?

0	*Never (0%)*
1	*Rarely (1-25%)*
2	*Some of the time (26-75%)*
3	*Most of the time (76-99%)*
4	*Always (100%)*

27.a) Have you ever had vaginal intercourse with your HITCH partner?

0	*No*
1	*Yes*

➡ ***If no, go to question 30.***

b) When did you first have vaginal intercourse with her? (If you only know the approximate date, specify the month and year.)

dd	*mm*	*yyyy*

c) When was the last time you had vaginal intercourse with her?

dd	*mm*	*yyyy*

d) Since the start of your sexual relationship with your HITCH partner, how many times did you have vaginal intercourse with her?

Approximate #	*OR*	*Approximate # times per week*	*OR*	*Approximate # times per month*

28. How often did you use condoms for vaginal intercourse with her? (This includes male and female condoms.)

0	*Never (0%)*
1	*Rarely (1-25%)*
2	*Some of the time (26-75%)*
3	*Most of the time (76-99%)*
4	*Always (100%)*

→ ***If never, go to question 30.***

29. **When you used condoms** for vaginal intercourse with your HITCH partner…

a) …did the condom ever break or slip off?

0	*No*
1	*Yes*
7	*Do not remember*

b) …did you always put the condom on before starting to have vaginal intercourse?

0	*No*
1	*Yes*
7	*Do not remember*

c) …did you ever take the condom off then continue to have unprotected vaginal intercourse?

0	*No*
1	*Yes*
7	*Do not remember*

30.a) Have you ever had anal intercourse with your HITCH partner?

0	*No*
1	*Yes*

→ ***If no, go to question 31.***

b) Since the start of your sexual relationship with your HITCH partner, how many times did you have anal intercourse with her?

	OR		*OR*	
Approximate #		*Approximate # times per week*		*Approximate # times per month*

c) How often did you use condoms for anal intercourse with her?

0	*Never (0%)*
1	*Rarely (1-25%)*
2	*Some of the time (26-75%)*
3	*Most of the time (76-99%)*
4	*Always (100%)*

Sexual Activity with Other Partners

The next questions are about sexual activities you may have engaged in with someone other than your HITCH partner.

31. Since the start of your relationship with your HITCH partner, did you engage in sexual activity with someone else? **Remember that by sexual activity, we mean mutual masturbation, oral, vaginal, and/or anal sex.**

0	*No*
1	*Yes*

➡ ***If yes, go to question 33.***

32. Is your HITCH partner the only partner with whom you have ever engaged in sexual activity **in your lifetime**?

0	*No*
1	*Yes*

➡ ***If yes, go to question 36 on page 16.***

➡ ***If no, complete one blue OP Form for the last person with whom you engaged in sexual activity before your HITCH partner,***
then go to question 36 on page 16.

<u>Do not answer questions 33-35.</u>

33. Since the start of your relationship with your HITCH partner, how many **other** sexual partners did you have?

Approximate #

→ ***If 5 or fewer other partners, complete a blue OP Form for each of these partners, then go to question 36 on page 16.***
<u>Do not answer questions 34-35.</u>

→ ***If more than 5 other partners, advance to question 34.***

34. Since the start of your relationship with your HITCH partner, how many **other** sexual partners were ongoing sexual partners? That is, partners with whom you had an **ongoing sexual relationship** (e.g. dating partner, wife, common-law partner)?

Approximate #

→ ***Complete a blue OP Form for each of these partners, then advance to question 35.***

35. Since the start of your relationship with your HITCH partner, how many **other** sexual partners were sexual partners with whom you did **<u>not</u> have an ongoing sexual relationship**? (e.g. one-night stands or flings)?

Approximate #

→ ***Complete one green AP Form for all of these partners combined, then advance to question 36.***

Medical History

The next questions refer to your medical history.

36. Did a doctor **ever** tell you that you had one of the following conditions?

				→ **If yes:** Were you told this since the start of your sexual relationship with your HITCH partner?		
	No	*Yes*	*Don't remember*	*No*	*Yes*	*Don't remember*
i) Trichomonas genital infection	*0*	*1*	*7*	*0*	*1*	*7*
ii) Venereal warts, condylomas, or papilloma virus infection	*0*	*1*	*7*	*0*	*1*	*7*
iii) Chlamydia	*0*	*1*	*7*	*0*	*1*	*7*
iv) Genital herpes	*0*	*1*	*7*	*0*	*1*	*7*
v) Syphilis	*0*	*1*	*7*	*0*	*1*	*7*
vi) Gonorrhea	*0*	*1*	*7*	*0*	*1*	*7*
vii) Ulcers or genital sores	*0*	*1*	*7*	*0*	*1*	*7*
viii) HIV	*0*	*1*	*7*	*0*	*1*	*7*
ix) Hepatitis B	*0*	*1*	*7*	*0*	*1*	*7*
x) Ureaplasma hominis	*0*	*1*	*7*	*0*	*1*	*7*

37. Since the start of your sexual relationship with your HITCH partner, did you have any of the following signs/symptoms?

	No	*Yes*	*Don't remember*
i) Painful urination, or difficulty urinating, or frequent urination	*0*	*1*	*7*
ii) Itching or burning sensation when urinating	*0*	*1*	*7*
iii) Blood in urine	*0*	*1*	*7*
iv) Abnormal discharge from penis	*0*	*1*	*7*
v) Sores in the genital area	*0*	*1*	*7*

Knowledge of HPV

This section is about your knowledge of and attitudes towards HPV. Please remember that you can speak with the Research Nurse after completing the survey if you have questions about HPV, penile cancer, or cervical cancer.

38. Before enrolling in the HITCH Cohort Study, had you ever heard of human papillomavirus, or HPV?

0	*No*
1	*Yes*

39. Please indicate whether the following statements are TRUE or FALSE.

		True	*False*	*Don't know*
i)	HPV can cause cervical cancer in women	*1*	*2*	*7*
ii)	Men can carry HPV	*1*	*2*	*7*
iii)	Genital warts cause cervical cancer in women	*1*	*2*	*7*
iv)	HPV can be cured with antibiotics	*1*	*2*	*7*
v)	A person may be infected with HPV and not know it	*1*	*2*	*7*
vi)	HPV can cause penile cancer in men	*1*	*2*	*7*
vii)	HPV causes genital herpes	*1*	*2*	*7*
viii)	Condoms protect against HPV	*1*	*2*	*7*
ix)	Having multiple sex partners increases one's risk for HPV	*1*	*2*	*7*
x)	Regular Pap tests can help to prevent complications from HPV	*1*	*2*	*7*
xi)	HPV is the most common sexually transmitted infection	*1*	*2*	*7*

40. Please indicate whether the following statements are TRUE or FALSE. A person can get HPV from…

		True	*False*	*Don't know*
i)	Sharing a plate, fork, or glass with someone who has HPV	*1*	*2*	*7*
ii)	Unprotected sexual intercourse with a someone who has HPV	*1*	*2*	*7*
iii)	Oral sex with someone who has HPV	*1*	*2*	*7*
iv)	Kissing (with exchange of saliva) someone who has HPV	*1*	*2*	*7*
v)	Sharing a washroom or shower with someone who has HPV	*1*	*2*	*7*

41. What do you think are your chances of becoming infected with HPV?

1	*Almost certain I will not*
2	*Very small chance*
3	*Some chance*
4	*Large or very large chance*
5	*Almost certain that I will get infected*
6	*I am already infected*

42. What do you think are your chances of developing penile cancer?

1	*Almost certain I will not*
2	*Very small chance*
3	*Some chance*
4	*Large or very large chance*
5	*Almost certain that I will get develop penile cancer*
6	*I have already been diagnosed with penile cancer*

HPV Vaccine

The last set of questions are about HPV vaccines. Although an HPV vaccine is not currently licensed for men, some men may have received it if they participated in a clinical trial of the vaccine. It may become available for all men in the future.

43.a) Have you received the HPV vaccine?

0	*No*
1	*Yes*
7	*Don't know*

→ ***If no or don't know, go to question 44.***

b) Did you receive the vaccine as part of participation in a clinical trial?

0	*No*
1	*Yes*
7	*Don't know*

c) How many injections of the HPV vaccine have you received, including booster shots?

#

d) When was your last injection of the HPV vaccine? (If you only know the approximate date, specify the month and year.)

dd	*mm*	*yyyy*

→ ***Go to question 45.***

44. If the HPV vaccine is offered to you in the future, how likely is it that you will choose to be vaccinated?

1	*Very likely*
2	*Somewhat likely*
3	*Neutral*
4	*Somewhat unlikely*
5	*Very unlikely*

45. Please use the space below if you have any additional information you feel would be important for us to know.

Please record the time you stopped filling out the survey here.

Time finished survey: ____________________

This brings us to the end of this survey. Please take a moment to review you answers in all sections of the questionnaire. Again, try to answer all questions. A good guess will be more useful to the study than leaving the question blank.

Thank you very much for your participation!
