# Supplementary material for: Human Papillomavirus Infection and Transmission Among Couples Through Heterosexual Activity (HITCH) Cohort Study: Protocol Describing Design, Methods, and Research Goals
Source: JMIR Res Protoc. 2019 Jan 16;8(1):e11284. doi: 10.2196/11284 (PMC6352011; doi:10.2196/11284)
Supplement: Multimedia Appendix 5 [file resprot_v8i1e11284_app5.pdf]

Multimedia Appendix 8. Biological specimens in the HITCH Cohort Study, 2005-2011.

	Baseline		Follow-up 1 (4 months)		Follow-up 2 (8 months)		Follow-up 3 (12 months)		Follow-up 4 (18 months)		Follow-up 5 (24 months)	
	Women	Men	Women	Men	Women	Men	Women	Men	Women	Men	Women	Men
Genital specimens	503/495	549/535	454/451	430/422	416/413	1	378/375	-	333/326	-	298/286	-
Blood specimens	503/482	549/538	454/438	430/422	416/405	1	378/368	-	333/322	-	298/282	-
Oral Specimens[a]	226/220	262/258	232/228	261/256	10[b]	3	17[b]	-	14[b]	-	13[b]	-
Hand specimens[a]	225/204	259/248	231/217	256/240	10[b]	1	16[b]	-	13[b]	-	12[b]	-

Data reported indicate the number of available specimens/number of participants.
[a]Specimens were only collected as of 2008, not since the beginning of the study.
[b]Samples obtained from already enrolled women who had brought a second or third male partner for whom biological specimens are included within baseline specimens.
