# Supplementary material for: Human Papillomavirus Infection and Transmission Among Couples Through Heterosexual Activity (HITCH) Cohort Study: Protocol Describing Design, Methods, and Research Goals
Source: JMIR Res Protoc. 2019 Jan 16;8(1):e11284. doi: 10.2196/11284 (PMC6352011; doi:10.2196/11284)
Supplement: Multimedia Appendix 6 [file resprot_v8i1e11284_app6.pdf]

Multimedia Appendix 9. Baseline characteristics of the HITCH Cohort Study population (N=502), Montreal, Quebec, 2005-2011.

	Women	Men	Couple
Mean Age, years (SD)	21.0 (2.1)	22.7 (3.5)	—
Birthplace, *n* (%)			—
Canada	341 (67.9)	318 (63.3)	
United States	39 (7.8)	48 (9.6)	
Other	122 (24.3)	136 (27.1)	
Ethnicity, *n* (%)			—
French Canadian	133 (26.5)	121 (14.1)	
English Canadian	176 (35.1)	207 (41.2)	
Black Canadian	8 (1.6)	13 (2.6)	
Other	182 (36.2)	159 (31.7)	
Missing	*3 (0.6)*	*2 (0.4)*	
Education, *n* (%)			—
High School, partial	1 (0.2)	14 (2.8)	
High School, completed	76 (15.1)	101 (20.1)	
College, partial	29 (5.8)	70 (13.9)	
College, completed	102 (20.3)	79 (15.7)	
University, partial	248 (49.4)	183 (36.5)	
University, completed	46 (9.2)	55 (11.0)	
Smoking, *n* (%)			—
Never smokers	377 (75.1)	348 (69.3)	
Former smokers	65 (13.0)	59 (11.8)	
Current smokers	60 (12.0)	94 (18.7)	
Missing	—	*1 (0.2)*	
Self-identity as exclusively heterosexual, *n*	447 (89)	488 (97)	—

(%)			
Median lifetime number of vaginal sex partners (range)	5 (0-40)	5 (0-56)	—
Median age at first vaginal intercourse, years (range)	17 (11-23)	17 (12-26)	—
Median duration of sexual partnership, months (IQR)	—	—	4.2 (2.8-5.3)
Median frequency of vaginal sex/week (IQR)	—	—	4.0 (2.6-6.0)
Condom use for vaginal intercourse, *n* (%)	—	—	
Never (0%)			47 (9.4)
Rarely (1%-25%)			136 (27.1)
Sometimes (26%-75%)			137 (27.3)
Most of the time (76%-99%)			88 (17.5)
Always (100%)			89 (17.7)
Missing			5 (1.0)
Oral sex on female, *n* (%)	—	—	
Never (0%)			23 (4.6)
Rarely (1%-25%)			89 (17.7)
Sometimes (26%-75%)			295 (58.8)
Most of the time (76%-99%)			92 (18.3)
Always (100%)			3 (0.6)
Oral sex on male, *n* (%)	—	—	
Never (0%)			11 (2.2)
Rarely (1%-25%)			73 (14.5)

Sometimes (26%-75%)			312 (62.2)
Most of the time (76%-99%)			98 (19.5)
Always (100%)			8 (1.6)
Engaged in anal sex, *n* (%)	—	—	127 (25.3)
Any HPV			
Genital specimens	282 (57.0)	280 (56.4)	
Oral specimens[a]	7 (3.2)	16 (7.2)	
Hand specimens[a]	78 (36.1)	74 (33.5)	
Any high-risk HPV types[b]			
Genital specimens	205 (41.4)	196 (39.5)	
Oral specimens	4 (1.8)	10 (4.5)	
Hand specimens	45 (20.8)	40 (18.1)	
Any low-risk HPV types[c]			
Genital specimens	182 (36.8)	190 (38.3)	
Oral specimens	3 (1.4)	7 (3.1)	
Hand specimens	45 (20.8)	44 (19.9)	
Any HPV6, 11, 16, 18			
Genital specimens	107 (21.6)	107 (21.6)	
Oral specimens	0	5 (2.2)	
Hand specimens	22 (10.2)	20 (9.0)	

[a]Specimens were only collected as of 2008, not since the beginning of the study.
[b]HPV16, 18, 31, 33, 35, 39, 45, 51, 52, 56, 58, 59, 66, 68, 73, and 82.
[c]HPV6, 11, 26, 40, 42, 53, 54, 55, 61, 62, 64, 67, 69, 70, 71, 72, 81, 83, 84, and 89.
